# Supplementary material for: Novel 3D Bioglass Scaffolds for Bone Tissue Regeneration
Source: Polymers (Basel). 2022 Jan 22;14(3):445. doi: 10.3390/polym14030445 (PMC8839207; doi:10.3390/polym14030445)
Supplement: Supplementary file 1 [file polymers-14-00445-s001.zip › polymers-1551239-supplementary.pdf]

polymers

MDPI

Supplementary data

Novel 3D Bioglass scaffolds for bone tissue regeneration

Evangelos Daskalakis [1], Boyang Huang [1], Cian Vyas [1], Anil Ahmet Acar [2], Ali Fallah [2], Glen Cooper [1], Andrew Weightman [1], Gordon Blunn [5], Bahhatin Koc [2,3,4] and Paulo Bartolo [1,6,*]

[1] Department of Mechanical, Aerospace and Civil Engineering, University of Manchester, Manchester M13 9PL, UK; evangelos.daskalakis@manchester.ac.uk (E.D.); boyang.huang@manchester.ac.uk (B.H.); glen.cooper manchester.ac.uk (G.C.); andrew.weightman@manchester.ac.u (A.W.); paulojorge.dasilvabartolo@manchester.ac.uk (P.B.)
[2] Integrated Manufacturing Technologies Research and Application Center, Sabanci University, Tuzla 34956 Istanbul, Turkey; aacar@sabanciuniv.edu (A.A.A); ali.fallah@sabanciuniv.edu (A.F.); bahattinkoc@sabanciuniv.edu (B.K.)
[3] SUNUM Nanotechnology Research Center, Sabanci University, Tuzla 34956 Istanbul, Turkey; aacar@sabanciuniv.edu (A.A.A); ali.fallah@sabanciuniv.edu (A.F.); bahattinkoc@sabanciuniv.edu (B.K.)
[4] Faculty of Engineering and Natural Sciences, Sabanci University, Tuzla 34956 Istanbul, Turkey; aacar@sabanciuniv.edu (A.A.A); ali.fallah@sabanciuniv.edu (A.F.); bahattinkoc@sabanciuniv.edu (B.K.)
[5] School of Pharmacy and Biomedical Sciences, University of Portsmouth, Portsmouth, PO1 2DT, UK; gordon.blunn@port.ac.uk Affiliation 1; e-mail@e-mail.com
[6] Singapore Centre for 3D Printing, School of Mechanical and Aerospace Engineering, Nanyang Technological University, 639798 Singapore
* Correspondence: paulojorge.dasilvabartolo@manchester.ac.uk; pbartolo@ntu.edu.sg;

```
G90 G94
G0 Z30
M99
M90 P3 D3
M95 P1
G0 X-7.563 Y-0.275
G0 Z-1.85
M97
F20 G1 X-7.563 Y-0.275 Z-1.85
X-7.566 Y0.935 Z-1.85
X-16.619 Y0.358 Z-1.85
X-16.664 Y0.714 Z-1.85
X-7.649 Y2.142 Z-1.85
X-7.798 Y3.343 Z-1.85
X-16.737 Y1.064 Z-1.85
X-16.839 Y1.408 Z-1.85
X-8.062 Y4.523 Z-1.85
X-8.452 Y5.669 Z-1.85
X-16.97 Y1.741 Z-1.85
X-17.127 Y2.063 Z-1.85
X-8.881 Y6.8 Z-1.85
X-9.366 Y7.908 Z-1.85
X-17.31 Y2.371 Z-1.85
X-17.519 Y2.663 Z-1.85
X-9.924 Y8.982 Z-1.85
X-10.531 Y10.028 Z-1.85
X-17.75 Y2.936 Z-1.85
X-18.004 Y3.189 Z-1.85
X-11.221 Y11.021 Z-1.85
X-12.033 Y11.918 Z-1.85
X-18.277 Y3.421 Z-1.85
X-18.569 Y3.629 Z-1.85
X-12.937 Y12.722 Z-1.85
X-13.909 Y13.442 Z-1.85
X-18.876 Y3.813 Z-1.85
X-19.198 Y3.97 Z-1.85
X-14.922 Y14.103 Z-1.85
X-15.98 Y14.691 Z-1.85
X-19.532 Y4.1 Z-1.85
X-19.875 Y4.202 Z-1.85
X-17.081 Y15.191 Z-1.85
X-18.218 Y15.605 Z-1.85
X-20.226 Y4.276 Z-1.85
```

Figure S1. Continuous path algorithm to fabricate the scaffolds.
